# Causal effects of gut microbiota on gout and hyperuricemia: insights from genome-wide Mendelian randomization, RNA-sequencing, 16S rRNA sequencing, and metabolomes

**DOI:** 10.1042/BSR20240595

**Published:** 2024-11-26

**Authors:** Xia Liu, Zhe Feng, Fenglian Zhang, Bo Wang, Zhijuan Wei, Nanqing Liao, Min Zhang, Jian Liang, Lisheng Wang

**Affiliations:** 1 Medical College, Guangxi University, Nanning 530004, China; 2 HIV/AIDS Clinical Treatment Center of Guangxi (Nanning) and The Fourth People’s Hospital of Nanning, Nanning 530023, China; 3 Department of Joint and Sports Medicine, Ruikang Hospital Affiliated to Guangxi University of Chinese Medicine, Nanning 530011, China; 4 Department of Gerontology, Nanning Social Welfare Hospital, Nanning 530004, China

**Keywords:** Gout, gut microbiota, Hyperuricemia, Mendelian randomization, multi-omics

## Abstract

Background: This study investigated the causal relationship between gut microbiota (GM), serum metabolome, and host transcriptome in the development of gout and hyperuricemia (HUA) using genome-wide association studies (GWAS) data and HUA mouse model experiments.

Methods: Mendelian randomization (MR) analysis of GWAS summary statistics was performed using an inverse variance weighted (IVW) approach to determine or predict the causal role of the GM on gout. The HUA mouse model was used to characterize changes in the gut microbiome, host metabolome, and host kidney transcriptome by integrating cecal 16S rRNA sequencing, untargeted serum metabolomics, and host mRNA sequencing.

Results: Our analysis demonstrated causal effects of seven GM taxa on gout, including genera of *Ruminococcus, Odoribacter*, and *Bacteroides*. Thirty eight immune cell traits were associated with gout. Dysbiosis of *Dubosiella, Lactobacillus, Bacteroides, Alloprevotella*, and *Lachnospiraceae_NK4A136_group* genera were associated with changes in the serum metabolites and kidney transcriptome of the HUA model mice. The changes in the gut microbiome of the HUA model mice correlated significantly with alterations in the levels of serum metabolites such as taurodeoxycholic acid, phenylacetylglycine, vanylglycol, methyl hexadecanoic acid, carnosol, 6-aminopenicillanic acid, sphinganine, p-hydroxyphenylacetic acid, pyridoxamine, and de-o-methylsterigmatocystin, and expression of kidney genes such as *CNDP2, SELENOP, TTR, CAR3, SLC12A3, SCD1, PIGR, CD74, MFSD4B5*, and *NAPSA*.

Conclusion: Our study demonstrated a causal relationship between GM, immune cells, and gout. HUA development involved alterations in the vitamin B6 metabolism because of GM dysbiosis that resulted in altered pyridoxamine and pyridoxal levels, dysregulated sphingolipid metabolism, and excessive inflammation.

## Introduction

Hyperuricemia (HUA) is a purine metabolic disorder characterized by high levels of uric acid in blood circulation, and is directly related with gout, a crystal-related joint disease [1]. The deposition of monosodium urate crystals in tissues such as joints and kidneys because of excessive accumulation of uric acid in the body causes gout [2,3]. The 2017 Gout Report White Paper reported 170 million hyperuricemiac patients in China. About 47% of these patients developed gout. The annual increase in gout cases in China was reported at 9.7% [4]. The current trend shows increasing incidence of HUA and gout in the younger individuals. Therefore, there is an urgent need to identify the pathogenetic mechanisms underlying the development of HUA and gout, which would subsequently promote discovery and optimization of effective therapies for HUA and gout.

The imbalance in gut microbiota (GM) is significantly associated with the development of several human diseases, including HUA [5]. In patients with HUA, the GM composition is altered and accompanied by reduced microbiota richness and diversity and relative lower abundance of Coprococcus [6]. However, contradictory reports exist regarding the GM composition in patients with HUA and gout [7,8]. These differences may be caused by changes in diet, smoking, and alcohol consumption [9,10]. The precise mechanistic details underlying the development of HUA and gout are lacking. Therefore, further studies are necessary to determine the molecular mechanisms by which the GM regulate the pathogenesis of HUA and gout.

There are several challenges for researching the association between gout and GM. Firstly, lack of comprehensive, multicenter clinical studies limits robust characterization of the relationship between gout and GM across diverse patient demographics. Secondly, the current knowledge regarding the relationship of GM composition and functionality is still rudimentary. Lastly, previous studies have not thoroughly investigated the causative relationship between GM and gout.

In this study, we used a Mendelian randomization (MR) approach to determine the causative relationship between the GM and gout. Furthermore, we assessed the impact of the gut microbiome on HUA and aimed to delineate potential microbial pathways using a rodent model of HUA. We used 16S rRNA sequencing and UHPLC/Q-Orbitrap-MS techniques to analyze cecal microbiota and plasma metabolomics, respectively, using the potassium oxonate-induced HUA mice. Furthermore, kidney RNA sequencing analysis was performed to verify the above results and further determine the relationships between gene functions and metabolic pathways. Overall, this study aimed to enhance the current understanding of the linkage between gut microbes and HUA, and further shed light on the importance of the gut bacterial metabolism in host biology.

## Methods

### Two-sample Mendelian randomization analysis

In the two-phase MR study, we analyzed the influence of GM on the genetically estimated risk of gout and evaluated the potential mediating role of the immune cells. The summary statistics for GM were extracted from the NHGRI-EBI GWAS Catalog (https://www.ebi.ac.uk/gwas/) with accession numbers ranging from GCST90027446 to GCST90027857. The gout-related data were obtained from the Finngen_R10_M13_GOUT dataset and included 9,568 gout cases and 262,844 controls. The information for 731 immune cell traits was obtained from the GWAS Catalog (Genome-wide association studies, https://gwas.mrcieu.ac.uk/) with accession numbers ranging from GCST0001391 to GCST0002121 [11]. The study population was European.

The single nucleotide polymorphisms (SNPs) were selected as instrumental variables (IVs) for the GM and immune cell traits using *P*-value of less than 1 × 10^−5^ as the threshold [11]. The SNPs related to gout were identified using a more stringent threshold *P*-value of less than 5 × 10^−8^. The genetic variants were clustered using a maximum squared correlation of R∧2 < 0.001 within a range of 10,000 kb [12]. Statistical analyses were performed using the ‘Two Sample MR’, ‘Variant Annotation’, and ‘ieugwasr’ packages from R version 4.3.1 (https://www.r-project.org). Among the five available methods for the MR analysis, namely, ‘MR Egger’ [13], ‘Weighted median’ [14], ‘Inverse variance weighted (IVW)’ [15], ‘Simple mode’ [16], and ‘Weighted mode’ [14], the IVW method was selected as the primary analytical tool because of its precision and robustness. *P*<0.05 was considered significant for linking the exposures to outcomes.

### Generation of the hyperuricemia model mice

Male specific pathogen free (SPF) Kunming mice (20–25 g) were obtained from Tianqin Biotechnology Co., Ltd. (Changsha, China) and acclimated for 1 week in the experimental animal room of Guangxi University at a temperature of 24 ± 2°C, humidity of 50 ± 5%, and a 12 h light/dark cycle. The mice received free access to diet and water. All the animal experimental procedures were performed strictly according to standards prescribed by the Chinese Society of Laboratory Animals and were approved by the Medical Ethics Committee of Guangxi University (Approval No. GXU-2023-0203). The animal experiments were performed at the Medical College of Guangxi University.

HUA and renal dysfunction were induced in mice with a high-purine diet and intraperitoneal injections of 200 μl potassium oxonate (PO) for seven consecutive days. The high-purine diet was prepared by regranulating mouse feed (100 g), yeast extract (40 g), and yeast ribonucleic acid (2 g) after melting. The PO was dissolved in 0.5% sodium carboxymethyl cellulose at a concentration of 52.5 mg/ml.

We randomly assigned 16 mice into the following two groups (8–10 mice per group): normal control group and PO-induced model group. The normal group mice were provided with a normal diet and intraperitoneal injections of 200 μl 0.5% sodium carboxymethyl cellulose. The model group mice were provided with a high-purine diet and intraperitoneal injections of 200 μl PO. After 7 days, blood was collected from the orbital sinus of mice after anesthesia with ether. The mice were then killed by cervical dislocation.

### Urine collection

After the 7-day treatment, the mice were transferred to a clean and empty cage individually to collect urine. The urine samples were centrifuged at 2000 × g for 10 min, and the supernatant was used to analyze the levels of uric acid and creatinine.

### Serum and tissue sample collection

Animals were anesthetized using diethyl ether at 1 h after the last treatment. Then, blood was collected from all the mice, and serum samples were prepared for analyzing uric acid and creatinine levels. After killing, kidney and liver tissues were harvested by dissection, quickly chilled on ice, and stored in liquid nitrogen for subsequent analysis.

### Untargeted metabolomics on plasma

#### UHPLC/Q-Orbitrap-MS conditions

Chromatography was performed using Ultra-high Performance Liquid Chromatography (UPLC) coupled with a hybrid Q-Exactive quadrupole-Orbitrap mass spectrometer. The separation was performed with a ACQUITY UPLC BEH C18 column (50 mm × 2.1 mm internal diameter (i.d.), 1.7 μm particle size; Thermo Scientific, MA, U.S.A.) that was maintained at 30°C. The mobile phase consisted of 0.1% formic acid in water (solvent A) and methanol (solvent B). The following gradient program was used for the separation: 0 to 2 min, maintained at 95% A; 2 to 13 min, decreased from 95% A to 0% A; 13 to 16 min, held at 0% A; 16 to 16.1 min, increased from 0% A to 95% A; 16.1 to 19 min, steady at 95% A. The flow rate was set at 0.30 ml/min with an injection volume of 2 μl. The autosampler was cooled to 4°C. Both positive (ESI+) and negative (ESI−) electrospray ionization modes were used for enhanced mass spectrometric detection across a mass range of m/z 200–2000 Da. The key operating conditions for the positive (ESI+) and negative (ESI−) ion modes were as follows: ion spray voltage, 3.0 kV; cone voltage, 25 V; sheath gas pressure, 35 psi; auxiliary gas flow, 10 psi; source temperature, 120°C; and nitrogen dry gas flow at 10 ml/min with an atomizer temperature of 350°C. Mass spectrometry data acquisition was performed in the MSE mode at a resolution of 70,000 for the initial scan and 17,500 for the subsequent scan.

#### Data processing and multivariate data analysis

Progenesis QI software (Nonlinear Dynamics, Newcastle upon Tyne, U.K.) was used for raw data processing and multivariate analysis, peak detection, alignment, and additional processing. The variables were defined by extracting the peak area from each sample and normalization was achieved based on the retention time and m/z values. Subsequently, two-dimensional data matrices were generated and imported into the SIMCA-P 14.1 (Umetrics AB, Umea, Sweden) for pattern recognition. Any zero values were eliminated based on the 80% rule and data were prepared for further statistical analyses. Principal component analysis (PCA) was performed to ascertain the distribution of metabolites in the serum samples. The orthogonal partial least squares discriminant analysis (OPLS-DA) models were constructed to identify differences between samples, including key differential metabolites within the extensive dataset. Model validation was performed through K-fold cross-validation, permutation tests, and ANOVA. Specifically, permutation tests were used to confirm reliability of the OPLS-DA models. The variable importance in projection (VIP) score was used to quantify importance of variable, with higher scores indicating greater contribution. VIP values were derived from the OPLS-DA model. The significant differential metabolites were defined by VIP>1, *t*-test *P*<0.05, and fold change (log2FC) ≥ 2.0 or ≤ 0.5.

#### Identification of metabolites and analysis of metabolic pathways

The structural characteristics of differential metabolites were determined using the human metabolome database (HMDB; http://www.hmdb.ca/) and METLIN (http://metlin.scripps.edu) based on the exact molecular mass, ionization method, and additional ion details. It was ensured that the m/z value deviation did not surpass 0.02. The metabolites were further validated by matching their exact charge number and ionization method to the experimental parameters. The differential metabolites associated with HUA were identified by comparing the primary and secondary mass spectral data of these metabolites against the theoretical fragmentation patterns from HMDB to determine their compound structures and assign the fragment origins.

The MetaboAnalyst 5.0 platform (http://www.metaboanalyst.ca) was used to determine alterations in major metabolic pathways associated with these differential metabolites. The impact values and *P*-values of the enriched pathways were presented in a bubble chart. The pathways demonstrating an impact greater than 0.2 and a *P*-value less than 0.05 were considered as significantly affected.

#### Evaluation of potential biomarkers

The potential biomarkers for HUA were assessed using the receiver operating characteristic (ROC) curve analyses with the GraphPad Prism software version 8.0. The sensitivity and specificity values were derived by adjusting the cut-off values. Then, we generated a plot of sensitivity on the *y*-axis against the false positive rate on the *x*-axis. These curves were used to calculate the area under the curve (AUC) value, which reflected the discriminative power of a biomarker between two distinct groups (e.g., experimental vs. control, or diseased vs. healthy). The AUC values typically range from 0.5 to 1.0. Higher AUC values suggested better predictive capability. Biomarkers with AUC values ≥ 0.9 are considered as highly accurate for predicting the disease state and were chosen as promising candidates for further investigation. Differential metabolites with AUC values ≥ 0.9 were considered as those significantly associated with HUA and their corresponding metabolic pathways. They were chosen for subsequent validation as candidate biomarkers.

#### Extraction of DNA and amplification of 16S rRNA

DNA was extracted from cecal samples and 16S rRNA amplification was performed as described previously [17].

#### Extraction of RNA and sequencing of RNA (RNA-seq)

RNA sequencing was performed by Novogene Co., Ltd using standardized procedures as previously reported [17]. Mean FPKM values were used to screen the differentially expressed genes, which were then analyzed by Gene Ontology (GO) and KEGG pathway enrichment analyses.

### Statistical analysis

SPSS 27.0 (SPSS, Chicago, IL, U.S.A.) was used for the statistical analyses. The data were represented as mean ± standard deviation. The statistical differences between groups were analyzed using the Student’s *t*-test. *P*-values <0.05 (*) were considered statistically significant. *P*-values <0.01 (**) were considered highly statistically significant. O2-PLS models were used to integrate the metabolome, transcriptome, and GM data sets [18]. O2-PLS models were calculated for the pair-wise data sets. Significant variables were then selected based on their correlation with the scores of the model (*P*<0.01).

## Results

### Mendelian randomization results

#### Total effect of gut microbiota on gout

**Figure 1:**
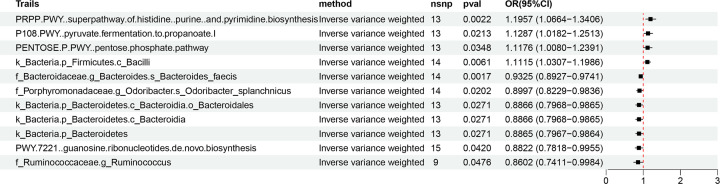
Mendelian randomization analysis shows significant association of 11 gut bacterial genera and metabolic pathways with gout CI: confidence interval; MR: Mendelian randomization; nsnp: single nucleotide polymorphism; OR: odds ratio.

A two-sample randomization analysis with the IVW method was performed to evaluate the causal relationship between GM and gout, and designated as the total effect. We identified 11 potential associations between the GM and gout (P_IVW_ < 0.05, FDR > 0.1; corresponding to 44 unique GM taxa). These bacterial genera potentially influenced the development of gout (Figure 1). Bacterial species such as *Bacteroides_faecis* (OR = 0.93, 95%CI: 0.89–0.97, *P* = 0.0017), and *Odoribacter_splanchnicus* (OR = 0.89, 95%CI: 0.82–0.98, *P* = 0.02), phylum *Bacteroidetes* (OR = 0.89, 95%CI: 0.79–0.98, *P* = 0.02), order *Bacteroidales* (OR = 0.88, 95%CI: 0.79–0.98, *P* = 0.02), genus *Ruminococcus* (OR = 0.86, 95%CI: 0.74–0.99, *P* = 0.04), and classes *Bacteroidia* (OR = 0.88, 95%CI: 0.79–0.98, *P* = 0.02), and PWY.7221 (OR = 0.88, 95%CI: 0.79–0.98, *P* = 0.04) showed protective effects against gout. In contrast, pyruvate fermentation to propanoate I (OR = 1.12, 95%CI: 1.01–1.25, *P* = 0.02), pentose phosphate pathway (OR = 1.11, 95%CI: 1.00–1.23, *P* = 0.03), super pathway of histidine purine and pyrimidine biosynthesis (OR = 1.19, 95%CI: 1.06–1.34, *P* = 0.02), and class *Bacilli* (OR = 1.11, 95%CI: 1.03–1.19, *P* = 0.006) showed positive association with the risk of gout. Therefore, up-regulation of pyruvate fermentation to propanoate, pentose phosphate pathway, and super pathway of histidine purine and pyrimidine biosynthesis was potentially associated with the development of gout.

#### Causal association between immune cell traits and gout

**Figure 2:**
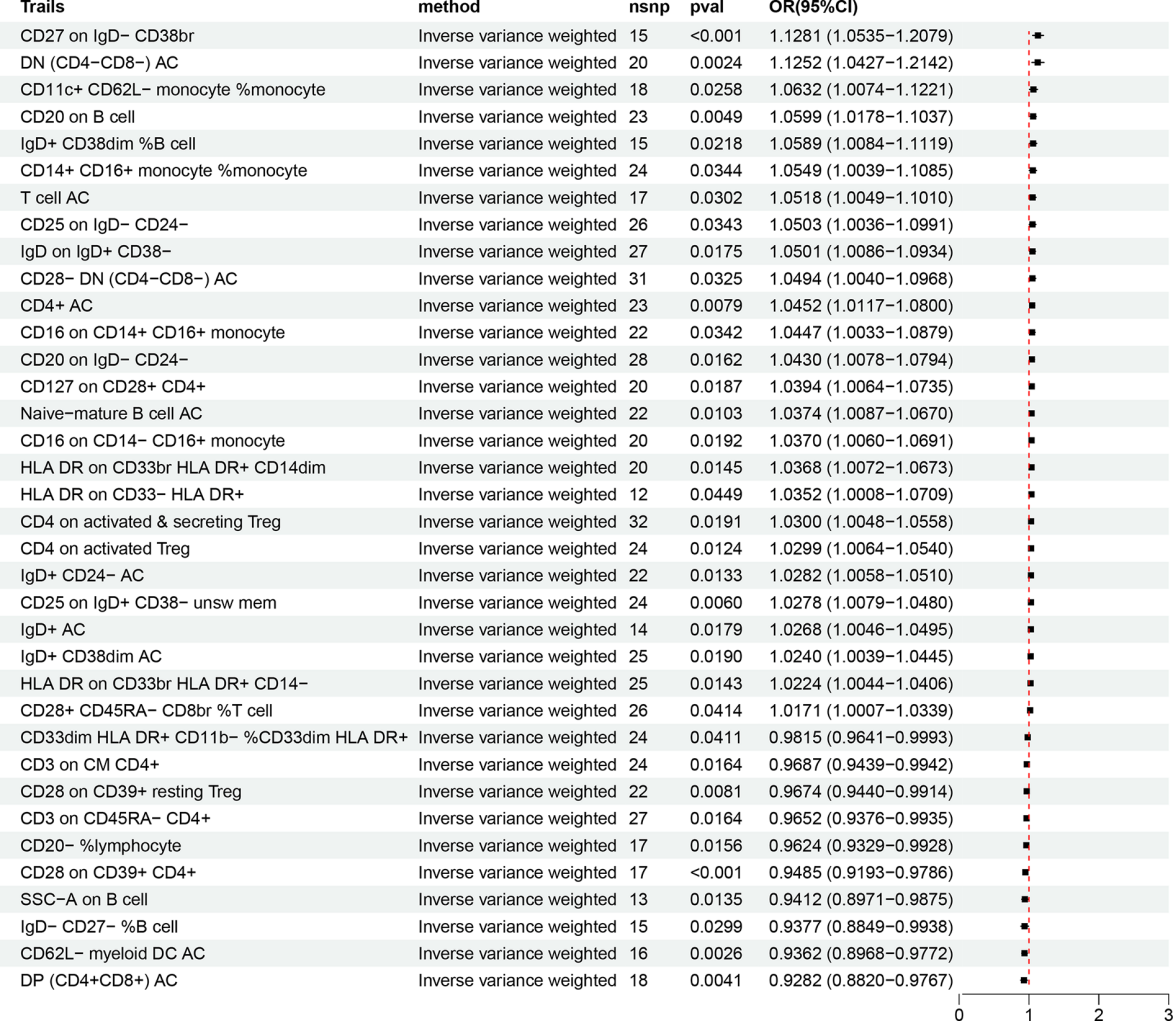
MR analysis shows causal relationships between immune cell traits and gout. As shown, 10 immune cell traits show protective effects against gout (OR<1) and 26 immune cell traits are associated with increased risk of gout (OR>1). CI: confidence interval; MR: Mendelian randomization; nsnp: single nucleotide polymorphism; OR: odds ratio.

To explore whether those 11 GM regulate the immune cell traits to affect gout, we conducted a two-step MR analysis. Based on the IVW method, 36 immune cell traits were associated with gout (Figure 2). This included 26 genetically-predicted immune cell traits associated with increased risk of gout and 10 immune cell traits associated with reduced risk of gout.

### Causal effects of gut microbiota on the immune cell traits

**Figure 3:**
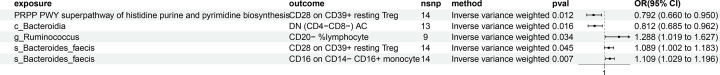
MR analysis shows significantly high association between four bacterial genera and five immune cell traits CI: confidence interval; MR: Mendelian randomization; nsnp: single nucleotide polymorphism; OR: odds ratio.

In previous studies, we identified 11 microbial taxa and 36 immune cell traits that play key roles in gout. We further explored the causal relationships between GM and immune cells. Using the IVW method, we found significant associations between four taxa and five immune cell traits (*P*<0.05, Figure 3). Specifically, *g.Ruminococcus* and *s.Bacteroides faecis* were positively associated with immune cell traits, whereas the PRPP PWY superpathway of histidine, purine, and pyrimidine biosynthesis and *C.Bacteroidia* exhibited negative associations with immune cell traits.

### Mediation effect of immune cells in the association between gut microbiota and gout

**Figure 4:**
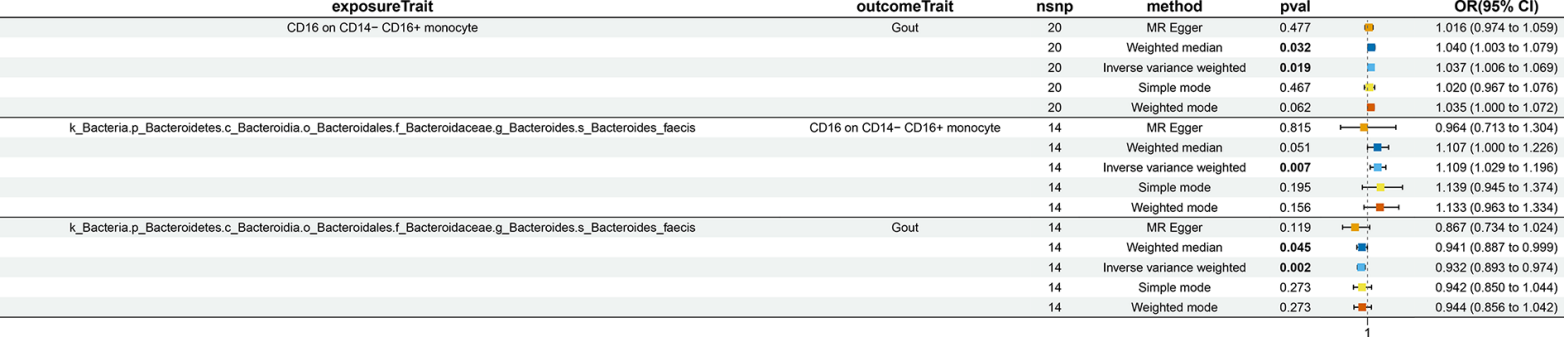
Reverse MR analysis results show the causal role of gout on Species-*Bacteroides_faecis* and CD16 on CD14− CD16+ monocytes, as well as, CD16 on CD14− CD16+ monocytes on Species-*Bacteroides_faecis* CI: confidence interval; MR: Mendelian randomization; nsnp: single nucleotide polymorphism; OR: odds ratio.

We analyzed the causal effects of Species-*Bacteroides_faecis* on gout and CD16 on CD14^−^ CD16^+^ monocytes. Subsequently, we analyzed whether CD16 on CD14^−^ CD16^+^ monocytes mediated the effects between Species-*Bacteroides_faecis* and gout (Figure 4). Our data showed that the mediation effect of CD16 on CD14^−^ CD16^+^ monocytes in the causal relationship between Species-*Bacteroides_faecis* and gout was 0.004 (95% CI: −0.004, 0.0117, Table 1), which accounted for −5.39% of the total effect.

**Table 1:** Mediation effect of *Bacteroides_faecis* on gout via CD16 on CD14^−^ CD16^+^ monocytes

Gut microbes	Immune cell trait	Outcome	Mediation effect
Species*-Bacteroides_faecis*	CD16 on CD14^−^ CD16^+^ monocytes	Gout	0.00377(−0.00413, 0.0117)

### Establishment of mouse model with abnormal uric acid levels

It is well known that sustained HUA causes gout [3]. In the present study, we established a mouse model of HUA based on a high-purine diet and intraperitoneal injections of PO to determine the molecular mechanisms by which GM influenced the development of gout and HUA. The serum and urine levels of uric acid in the control and model group mice are shown in Tables 2 and 3. Our data showed that the serum and urine levels of uric acid were significantly higher in the HUA model group compared with the normal group (*P*<0.05). This indicated that the HUA mouse model was established successfully. Furthermore, the serum levels of creatinine and blood urea nitrogen also increased in the HUA model group mice. Moreover, urine creatinine levels decreased in the model mice compared with the control group mice, but the differences were not statistically significant.

**Table 2:** Serum parameters in the hyperuricemia model and control group mice

Parameters	*n*	Normal group	HUA model group	*P-*value
BUN	5	13.069±2.898	20.193±14.322	*0.091*
Creatinine	5	42.890±4.119	237.522±148.167	*0.039*
Uric acid	10	111.655±12.011	212.401±8.232	*<0.0001*

**Table 3:** Urine parameters in the hyperuricemia model and control group mice

Parameters	*n*	Normal group	HUA model group	*P*-value
creatinine	5	2160.579±223.538	1991.113±169.962	*0.637*
Uric acid	10	543.927±99.174	984.872±40.460	*0.008*

### Diversity and functional assessment of gut microbiota in the hyperuricemia model mice

**Figure 5:**
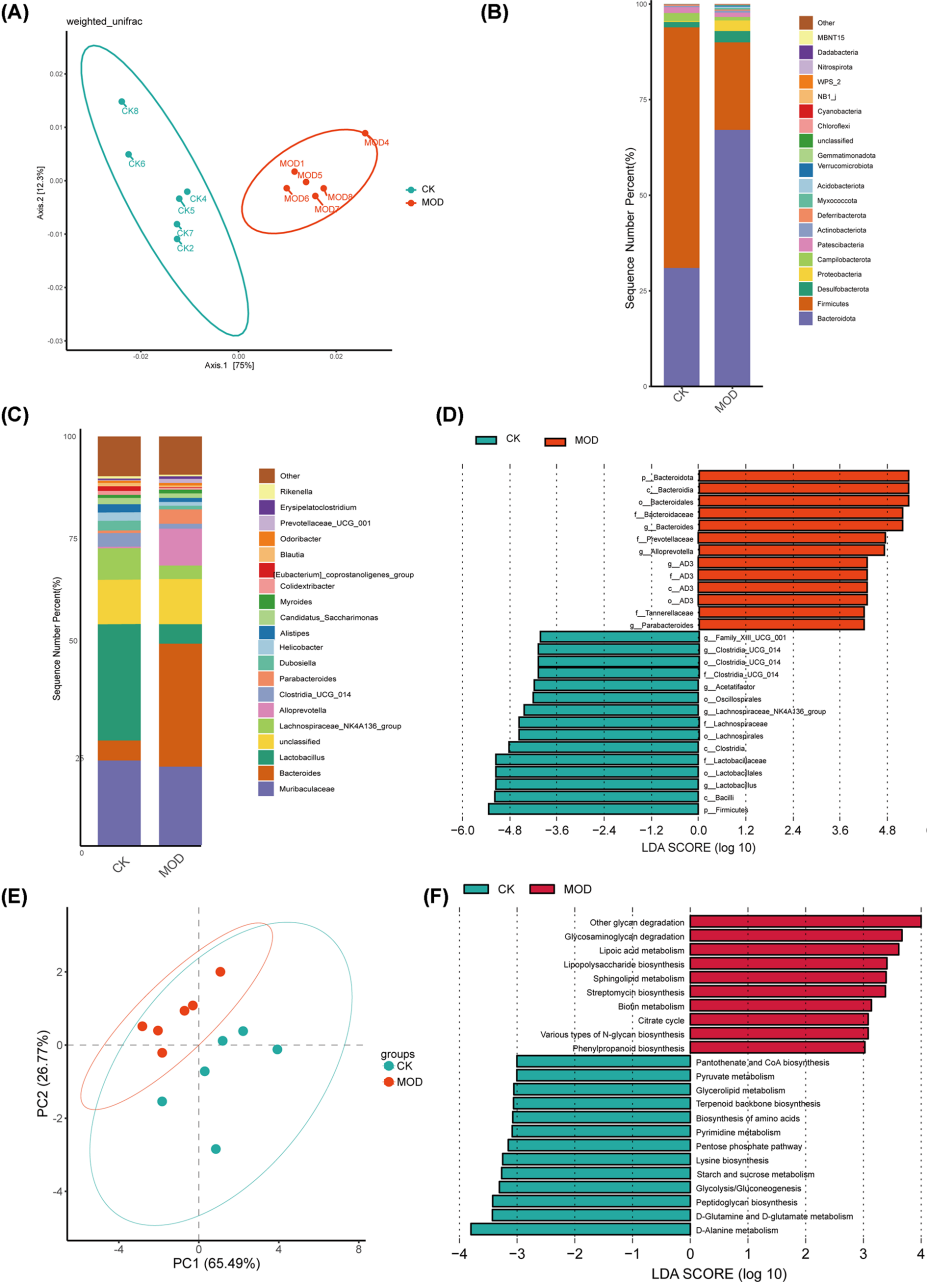
Hyperuricemia induction causes taxonomic and functional dysbiosis in the colon microbiome (**A**) PCoA analysis plot shows gut microbial beta diversity in the control (blue dots; CK) and HUA model (red dots; MOD) groups. Each dot represents an individual mouse. (**B**) The distribution plot shows relative abundance of gut bacteria at the phylum level in the control (CK) and HUA model (MOD) groups. (**C**) The distribution plot shows relative abundance of bacteria at the genus level in the control (CK) and HUA model (MOD) groups. (**D**) LefSe analysis results show differentially abundant gut bacterial taxa between the control (CK) and HUA model (MOD) groups. (**E**) PCoA analysis shows relative differences in the functional signatures between the gut microbiomes in the control and HUA model mice. Each dot represents an individual mouse. (**F**) Overview of KEGG metabolic pathway profiles in the gut microbiomes of the control and HUA model groups of mice. The relative abundances in (**B–D**) and (**F**) were calculated based on the mean values from six mice per group. CK: control group; MOD: HUA model group.

To explore the role of GM in the pathogenesis of HUA, we performed cecal 16S rRNA sequencing analyses. The α-diversity between the HUA model and control groups was evaluated using the Chao1, Shannon, and Simpson indices. We did not observe any significant differences in the α-diversity between the control and HUA model groups (Supplementary Table S1). However, beta diversity analysis showed that the phylogenetic community structures were significantly different between the normal control and the HUA model samples (Figure 5A), as evaluated by the principal coordinate analysis (PCoA). This suggested significant alterations in the GM composition between the HUA model and control groups and were consistent with the MR analysis. This further demonstrated that structural changes in the GM played a critical role in the development of HUA [10].

The GM was dominated by phyla such as *Bacteroidetes, Firmicutes, Desulfobacterota, Proteobacteria*, and *Patescibacteria*. We observed significant difference in these phyla between the control group and HUA model groups. The community bar plot in Figure 5B shows the top 20 most abundant phyla. The HUA model group showed significant reduction in the *Firmicutes* phylum and significant increase in the *Bacteroidota* phylum compared with the control group. Therefore, the *Firmicutes* to *Bacteroidota* ratio was significantly reduced in the HUA model group compared with the control group. This demonstrated association between HUA and GM dysbiosis.

The relative abundances of various genera are shown in Figure 5C. The HUA model group mice showed significant increase in the abundances of three bacterial taxa (*Bacteroides, Alloprevotella*, and *Parabacteroides*) and significant reduction in the abundances of five bacterial taxa (*Lactobacillus, Lachnospiraceae_NK4A136_group, Clostridia_UCG_014, Muribaculaceae*, and *Dubosiella*) compared with the control group. LEfSe analysis was performed to compare the differences between the control and HUA model groups at the genus level. Based on the threshold parameters such as logarithmic LDA score > 4.0 and *P*<0.01, LefSe analysis results showed that the *Bacteroides, Alloprevotella*, and *Parabacteroides* genera were enriched in the model group, whereas *Clostridia_UCG_014, Acetatifactor, Lachnospiraceae_NK4A136_group*, and *Lactobacillus* genera were enriched in the control group (Figure 5D).

PICRUST2 software analysis predicted 382 differentially expressed KEGG pathways, including metabolism (43.3%), organismal systems (18.7%), human diseases (19.6%), genetic information processing (5.5%), environmental information processing (8.3%), and cellular processes (4.7%). We focused on the link between metabolic pathways and HUA pathophysiology. To detect the key metabolic pathways involved in the development of HUA, we performed LEfSe analysis and RF analysis between the control and HUA model groups. Based on the logarithmic LDA score of 3.0 as the cut-off, we identified 23 significantly different metabolic pathways that were related to HUA (Figure 5E,F). Based on the RF analysis plot (MDA score >2.0) (Supplementary Table S2), we identified 13 key pathways associated with HUA. Furthermore, our data showed that the vitamin B6 metabolism was associated with the occurrence and development of HUA.

### Broad changes in the serum metabolome of the hyperuricemia model group mice

**Figure 6:**
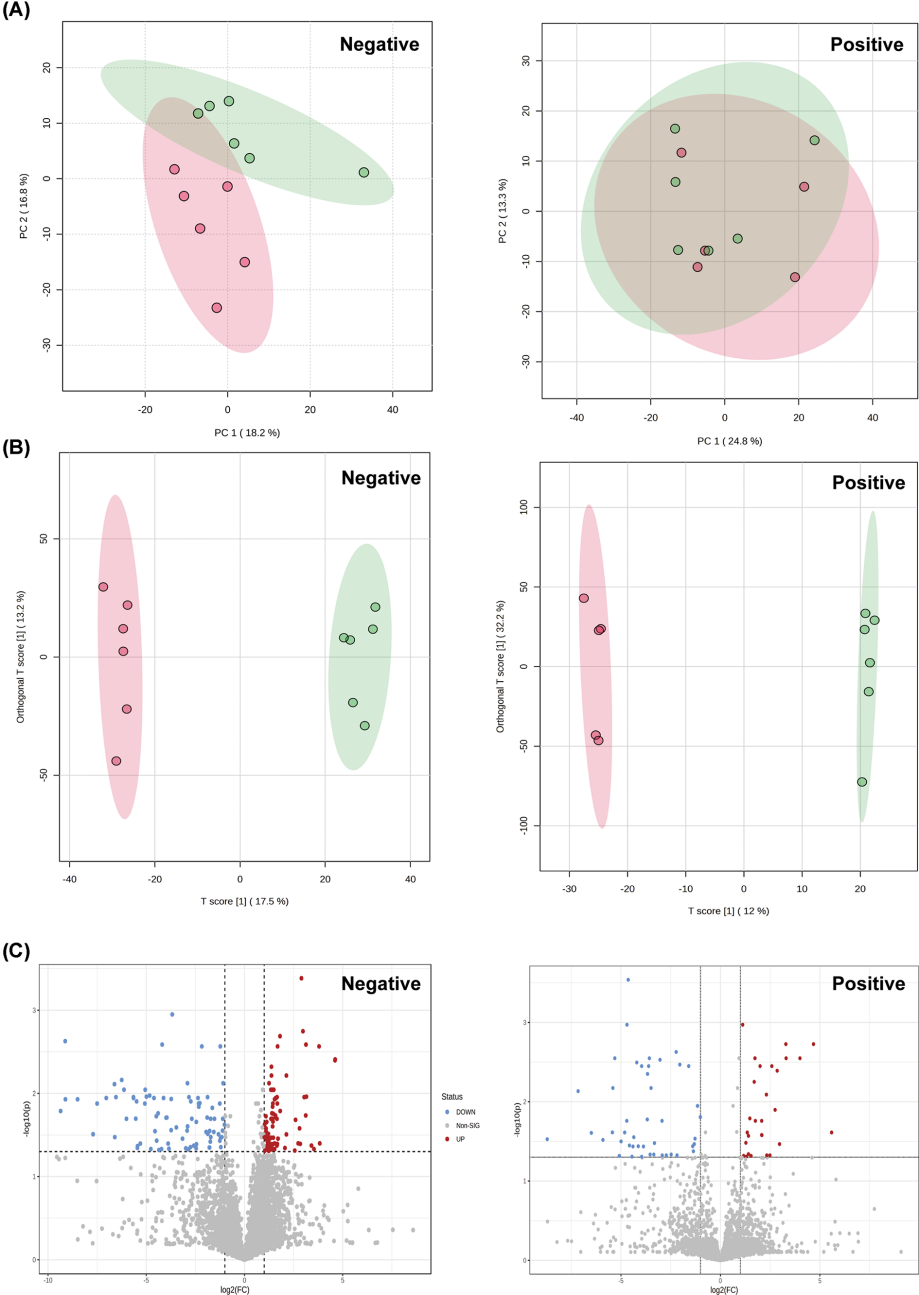
Serum metabolite changes in the hyperuricemia model group (**A**) PCA score plots of the control (red dots; CK) and HUA model (green dots; MOD) groups in the positive and negative ion modes. (**B**) OPLS-DA score plots of the control (red dots; CK) and HUA model (green dots; MOD) groups in the positive and negative ion modes. (**C**) Volcano plot shows the most significant and differentially expressed metabolites (red and blue dots, MOD vs. CK), as identified by the univariate analysis. CK: control group; MOD: HUA model group.

To fully characterize the impact of altered GM composition and functional dysbiosis, we performed untargeted metabolomics of the murine serum samples from the HUA model and control groups. Multivariate statistical analysis was performed using PCA, partial least squares-discriminant analysis (PLS-DA) models, and OPLS-DA models, and the results are shown in Figure 6. PLS-DA and OPLS-DA models demonstrated significant separation between the HUA model and control groups. Compared with the unsupervised PCA model, the supervised OPLS-DA model showed superior performance in distinguishing between the HUA and control groups, and identify differential markers. We observed distinct separation of the HUA model group and the control group clusters. This demonstrated that the induction of the HUA model caused significant changes in the levels of endogenous metabolites. The volcano plot in Figure 6C shows metabolite changes caused by the HUA model group compared with the control. Subsequently, significantly differentially expressed metabolites were selected using |log_2_FC|≥2 and *P*<0.05 as threshold parameters. We identified 68 serum metabolites with significant changes in their expression levels in HUA model group, based on previous studies and data obtained from online databases (Supplementary Table S3).

Metabolic pathway analysis of all the differentially expressed metabolites (*n* = 68) was performed using the MetPA tool and the results are presented in the form of an interactive visualization system (Supplementary Table S4). The metabolic pathway enrichment and topological analysis results demonstrated that the HUA pathogenesis in mice was mainly associated with metabolic pathways related to androgen and estrogen metabolism, tryptophan metabolism, and vitamin B6 metabolism.

### Kidney transcriptometric analysis of the hyperuricemia model group mice

**Figure 7:**
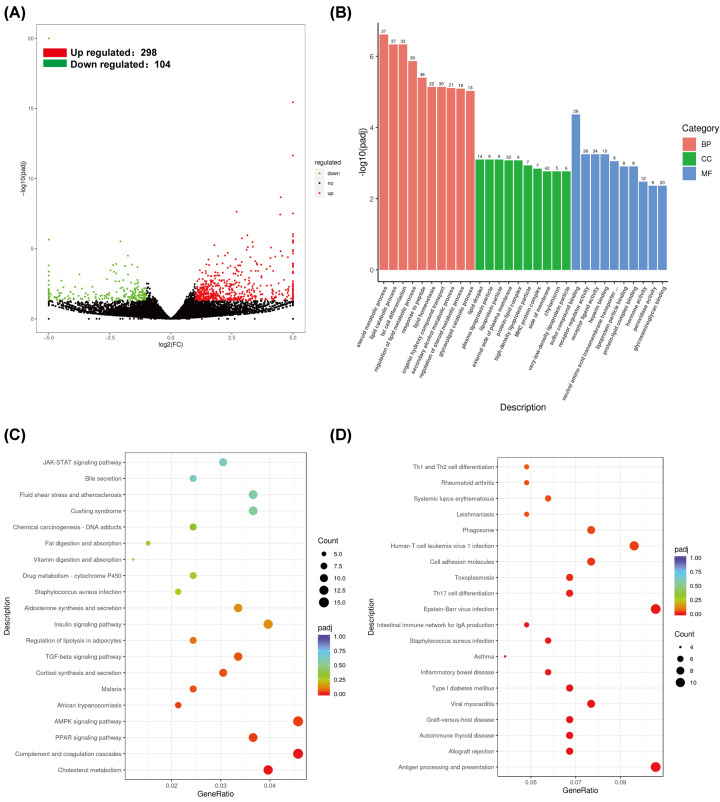
Global overview of the kidney transcriptome changes in the hyperuricemia model mice (**A**) Volcano plot shows differential expressed genes (DEGs) between the HUA model group and the control group with Log2 (Fold change) >2 and *P*<0.05 as threshold parameters. (**B**) GO annotation enrichment analysis of the DEGs, performed using clusterProfiler 3.8.1. (**C,D**) KEGG pathway enrichment analysis of 298 up-regulated and 104 down-regulated DEGs, conducted using clusterProfiler 3.8.1.

To determine the genome-wide impact of HUA-related gut dysbiosis on the host transcriptome, we compared the kidney mRNA expression levels in the control and HUA model groups. Kidney RNA-seq analyses demonstrated up-regulation of 298 differential expressed genes (DEGs) and down-regulation of 104 DEGs in the control group compared with the HUA model group (Figure 7A and Supplementary Table S5). GO analysis of the DEGs showed enrichment of biological processes such as steroid metabolic process, lipid catabolic process, and fat cell differentiation; molecular functions such as sulfur compound binding, receptor regulator activity, and receptor ligand activity; and cellular components such as lipid droplet, plasma lipoprotein particle, and external side of plasma membrane (Figure 7B). Furthermore, KEGG pathway analysis results showed that up-regulated DEGs were enriched in pathways such as AMPK signaling pathway, complement and coagulation cascades, and cholesterol metabolism, whereas, down-regulated DEGs were enriched in pathways such as antigen processing and presentation, intestinal immune network for IgA production, and Th1 and Th2 cell differentiation. Taken together, the comparative analysis of the kidney gene expression patterns between the control and HUA model groups demonstrated significant dysregulation of metabolic process and reduced immune response after PO treatment-induced HUA.

### Systematic molecular correlations across multiomes integrating microbiome, metabolome, and host transcriptome

**Figure 8:**
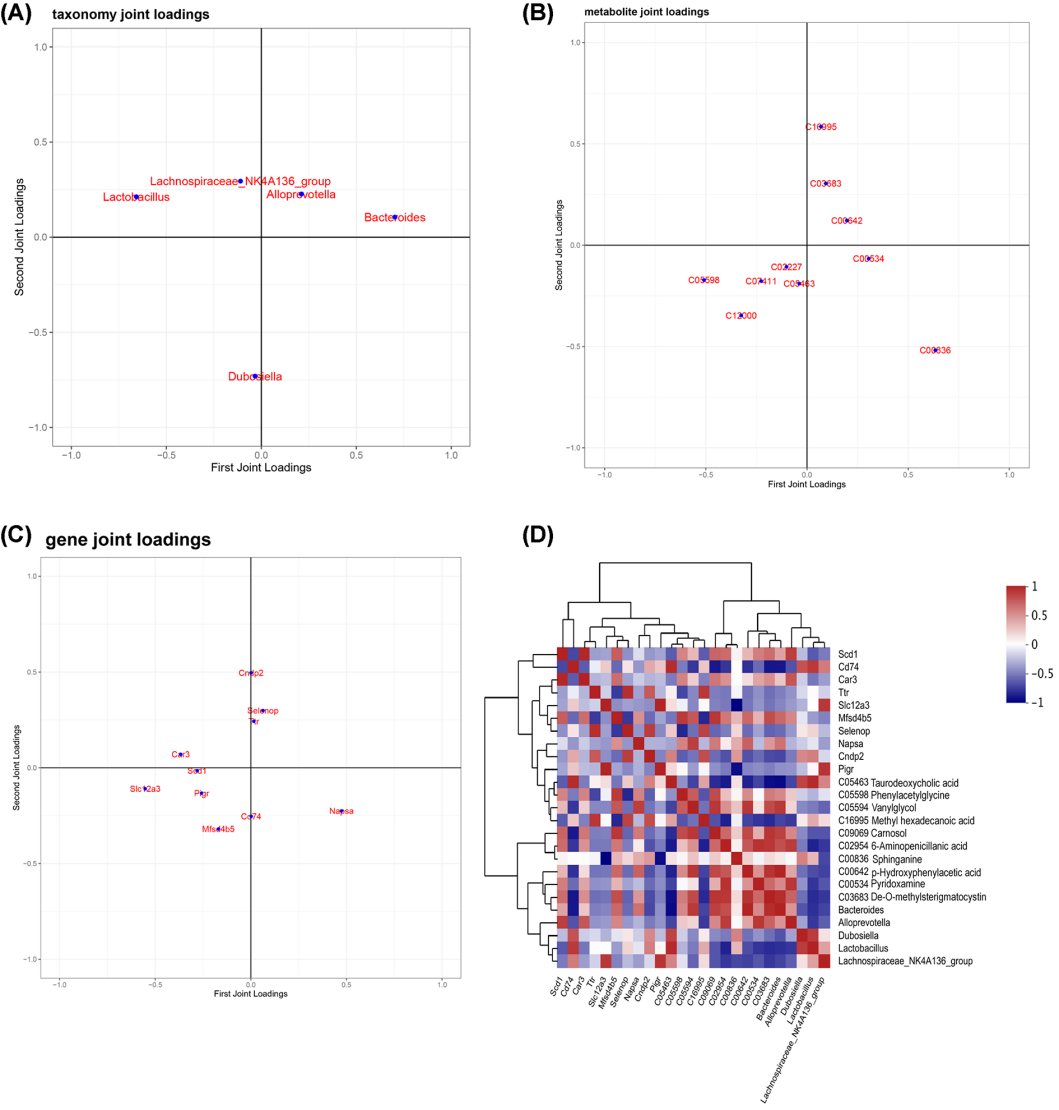
Microbiota–metabolite–transcriptome relationships (**A**) O2PLS-DA load diagram of the GM (**B**) O2PLS-DA load diagram of the serum metabolites. (**C**) O2PLS-DA load diagram of the differentially expressed genes. (**D**) Spearman correlation analysis of key GM, serum metabolites, and differentially expressed genes.

During disease progression, changes in metabolic profiles coincide with changes in the transcriptome and the abundances of microbial communities. To determine this relationship, we used O2-PLS statistical modeling to determine significant correlations between microbial groups and serum metabolic profiles, as well as between the serum metabolic profiles and the kidney transcriptome. Data from the UHPLC-MS spectra, RNA-seq, and 16S rRNA sequencing analysis of absolute gut microbial abundances were analyzed using the O2-PLS regression models to determine meaningful statistical and biological correlations among the microbial taxa in the colon, kidney transcriptome, and specific serum metabolites. The O2-PLS models showed that *Dubosiella, Lactobacillus, Bacteroides, Alloprevotella*, and *Lachnospiraceae_NK4A136_group* were the most significantly altered genera during HUA development in the mouse model (Figure 8A). Furthermore, the O2-PLS analysis demonstrated significant correlations between these gut bacterial genera and serum metabolites such as taurodeoxycholic acid, phenylacetylglycine, vanillylglycol, methyl hexadecanoic acid, carnosol, 6-aminopenicillanic acid, sphinganine, p-hydroxyphenylacetic acid, pyridoxamine, and de-O-methylsterigmatocystin (Figure 8B). Moreover, O2-PLS models also demonstrated significant correlation between the serum metabolome and the expression levels of genes such as *CNDP2, SELENOP, TTR, CAR3, SLC12A3, SCD1, PIGR, CD74, MFSD4B5*, and *NAPSA* from the kidney transcriptome (Figure 8C).

Furthermore, to delineate both direct and indirect microbial–host interactions related to HUA, an interaction heatmap between the three omics datasets was constructed using Spearman correlation analysis (Figure 8D and Supplementary Table S6). Specifically, sphinganine showed positive correlations with genes involved in the sphingolipid metabolism pathway and pyridoxamine demonstrated significant negative correlations with genes related with vitamin B6 metabolism.

## Discussion

In this study, we performed MR analysis to determine the association between GM dysbiosis and gout. Next, we established a rodent model of HUA to validate the role of GM and identify the GM-regulated metabolites and genes involved in the pathogenesis of gout and HUA. Based on the initial results of the MR analysis, we performed in-depth investigation of the role of the gut microbiome in the pathogenesis of HUA. We identified several bacteria that alter host metabolism and a cluster of metabolites that influence the pathogenesis of HUA. Altogether, this study has implications beyond the effect of GM on HUA. It provides insights into the regulation of uric acid levels in circulation by the gut bacterial metabolism. Overall, our data suggested that alteration of host metabolism by gut microbes played a significant role in the development of HUA and gout.

We performed a two-sample MR to assess the causal effects of GM on gout and identified 11 microbial taxa that showed a significant causal relationship with gout. This included species such as *Bacteroides_faecis* and *Odoribacter_splanchnicus*, phylum *Bacteroidetes*, order *Bacteroidales, Ruminococcus* genera, class *Bacteroidia*, PWY.7221, pyruvate fermentation to propanoate I, pentose phosphate pathway, super pathway of histidine purine and pyrimidine biosynthesis, and class *Bacilli*. The GM influenced the pathogenesis of HUA and gout by altering the normal regulation of the immune system and host metabolism. Furthermore, we performed a two-step MR to determine if these 11 taxa impacted gout by modulating the immune system. Preliminary data identified 26 protective immune cell traits against gout and 12 risk-promoting immune cell traits. Subsequent analysis of the causal relationship between the 11 bacterial taxa and 36 immune cell traits demonstrated a significant association between *Bacteroides_faecis* and CD16 on CD14^−^ CD16^+^ monocytes. This suggested that CD16 on CD14− CD16+ monocytes was a potential and essential mediator that orchestrated the protective role of *Bacteroides_faecis* against gout. Our hypothesis is further supported by a two-step MR analysis using the IVW method, indicating a potential role of CD16^+^ monocytes in immune regulation. While CD16^+^ monocytes have been implicated in immune responses, further research is needed to confirm their involvement in inflammation modulation. *Bacteroides_faecis* may exert its protective effect by modulating these monocytes, which could play a role in inflammatory diseases.

HUA is considered as the early and essential stage of gout, which is primarily caused by an abnormal increase in the uric acid levels. Therefore, we generated a mouse model of HUA to validate the causal effects of GM on gout and HUA. Basic characteristics and biochemical test results demonstrated that the uric acid, creatinine, and BUN levels were significantly increased in the serum of the HUA model mice compared with the control group. Furthermore, the urinary uric acid levels were also significantly higher in the HUA model mice, but the urine creatinine levels were slightly lower and not statistically significant. These results suggested successful establishment of the HUA model mice. This model has been reported previously [19], and was optimized in our laboratory.

Detailed analysis on the diversity of the GM showed that the relative abundances of genera belonging to *Bacteroides, Alloprevotella*, and *Parabacteroides* were significantly increased and the relative abundances of genera belonging to *Lactobacillus, Lachnospiraceae_NK4A136_group, Clostridia_UCG_014, Muribaculaceae*, and *Dubosiella* were significantly decreased in the HUA model mice. Previous studies have reported comparable gut microbial dysbiosis across a range of metabolic disorders [7,10,20]. In our investigation, significant reduction in the relative abundance of *Firmicutes* within the HUA group mice contributed to further gut microbial imbalance, deterioration of the intestinal lining, reduced immune modulation, and disease progression. *Firmicutes* and *Bacteroidetes* are the two main phyla of the healthy adult intestinal microbiota, covering approximately 90% of known GM [21]. *Firmicutes* mainly include the most studied genera are *Faecalibacterium, Clostridium, Eubacterium, Roseburia, Blautia, Lactobacillus*, and *Ruminococcus*. It is reported that the genera of *Faecalibacterium spp*., *Eubacterium spp*., and *Roseburia spp*. are the representative butyric acid-producing bacteria, which could reverse the disorder of glucose and lipid metabolism, as well as ameliorate intestinal inflammation [22–25], and the lactic acid-producing bacteria represented by *Lactobacillus spp*. have a beneficial effect on obesity [24,26–29]. *Lactobacillus* facilitates breakdown and removal of uric acid (UA) in the gut through the production of short-chain fatty acids (SCFAs) [30]. Previous findings showed reduced abundance of *Lactobacillus, Streptococcus*, and *Clostridium*, which regulate purine metabolism and UA breakdown, in the intestinal flora of the HUA model rats, whereas, increased relative abundance of *Proteus* correlated with the production of xanthine dehydrogenase (XDH) [31]. Enrichment of *Desulfovibrio* and *Parabacteria* in gout is directly related to HUA [10]. Align with this, an elevation of *Desulfovibrio* and *Parabacteria* was also found in the HUA model group in the present study. However, the potential mechanisms by which *Desulfovibrio* and *Parabacteria* affect host health remain unclear. Currently, many studies suggest the mechanisms are mainly attributed to the metabolites that the bacteria own cellular components and the bacteria produce (especially hydrogen sulfide, H_2_S) [32]. H_2_S has been shown to mediate the expression of FXR, CYP7A1, and cholesterol transporter protein ABCG5/G8, ultimately affecting the balance of metabolism in the body [33]. Moreover, the reduction of *Lactobacillus* and the increase in *Bacteroides* were significantly associated with host metabolism and kidney gene expression, highlighting their roles in the pathogenesis of HUA. As shown in Figure 5(C), the abundance of *Lactobacillus* significantly decreased in the HUA model group, and a similar decrease was observed in a fructose-induced HUA model, leading to metabolic disturbances and intestinal barrier dysfunction. This reduction has been associated with decreased SCFA production, negatively affecting uric acid metabolism and contributing to disease progression [34]. The imbalance of these key bacteria may exacerbate gut dysbiosis and metabolic dysfunction, leading to further intestinal barrier impairment and disease exacerbation.

Metabolomic analysis on the host serum showed that the abnormal metabolites were enriched in metabolic pathways of tryptophan metabolism, steroid hormone biosynthesis, vitamin B6 metabolism. Previous studies have shown that the levels of sex hormones are associated with the occurrence and development of HUA via regulation of inflammation and metabolism related with gout [35–37]. Previous epidemiological data have shown that the incidence of HUA is 8.6% among females and 21.6% among males [38]. In the steroid hormone biosynthesis pathway, pregnenolone, an immunomodulatory metabolite, was significantly elevated in our study (log2FC = 2.33, *P*<0.05), consistent with reports linking pregnenolone increases to gout [39,40]. These findings underscore the importance of steroid hormone metabolism in HUA and gout pathogenesis.

Furthermore, the differentially expressed metabolites of N-acetylserotonin, 3-hydroxykynurenamine, 6-hydroxymelatonin, and 4-Hydroxyphenylacetate, enriched in the tryptophan metabolism pathway, were also decreased in HUA model group. Abnormalities in tryptophan and tyrosine metabolism have been associated with gout and HUA [41]. To our knowledge, this is the first report of altered levels of these metabolites in an HUA mouse model.

Additionally, a reduction in pyridoxamine and pyridoxal, forms of vitamin B6, was observed in the HUA model group. As we know, these two metabolites were forms of vitamin B6. Previous studies suggest that vitamin B6 supplementation lowers serum uric acid levels, although the exact mechanism remains unclear. Since vitamin B6 cannot be synthesized by the body, it is obtained from diet and bacterial synthesis via intestinal absorption [42]. Thus, normal gut function and a balanced microbiota are crucial for maintaining vitamin B6 homeostasis in the body. Vitamin B6 absorption is pH-dependent, with higher uptake observed under acidic conditions [43]. This aligns with our microbiome analysis, which showed a significant decrease in acidogenic bacteria, such as *Lactobacillus, Lachnospiraceae_NK4A136_group*, and *Clostridia_UCG_014* in HUA mice. The more alkaline environment in the gut of hyperuricemic model mice might cause decreased absorption of vitamin B6. This is consistent with previous research showing that vitamin B6 supplementation decreases uric acid levels [44]. These findings suggest that disturbances in vitamin B6 metabolism may be linked to gut microbiome imbalances contributing to HUA.

Subsequently, transcriptomics analysis of the kidney tissue samples identified in the HUA model mice that were significantly enriched in 35 KEGG pathways, including vitamin digestion and absorption, retinol metabolism, and cortisol synthesis and secretion. In our metabolomic analysis, it was also shown that differentially expressed serum metabolites were enriched in the vitamin B6 metabolism pathway. Furthermore, the down-regulated genes were predominantly associated with immune function, including differentiation of Th1, Th2, and Th17 cells, as well as the immune network responsible for IgA synthesis. These data were consistent with the previous MR analysis, which showed 36 immune cell traits play a role on gout. It seems that the transcriptomics analysis correspond with the above metabolome and MR analysis. Consistent with this, a research in a *Uox*-KO mice model also showed that impairment of intestinal integrity and alterations in the profile of solute carrier family, subsequently affected serum uric acid level and CD4+ Th17 driven inflammation [45]. Furthermore, another study demonstrated that HUA-induced gut dysbiosis contributes to the development of renal injury, possibly through production of gut-derived uremic toxins and subsequently activating the NLRP3 inflamasome [46]. Thus, we can concluded from the above results that HUA-induced GM dysbiosis contributes to the production of gut-derived metabolites, and subsequently promoting the inflammation in kidney.

Furthermore, O2-PLS regression-model was used to determine the GM–metabolite–transcriptome correlation network. This demonstrated that the genera belonging to *Dubosiella, Lactobacillus, Bacteroides, Alloprevotella* and *Lachnospiraceae_NK4A136_group* were associated with changes in the host metabolites and differential expression of the host genes during HUA development. During this process, metabolites such as taurodeoxycholic acid, phenylacetylglycine, vanylglycol, methyl hexadecanoic acid, carnosol, 6-aminopenicillanic acid, sphinganine, p-hydroxyphenylacetic acid, pyridoxamine, and de-o-methylsterigmatocystin, and genes such as *CNDP2, SELENOP, TTR, CAR3, SLC12A3, SCD1, PIGR, CD74, MFSD4B5*, and *NAPSA* were associated with alterations in the gut microbiome during HUA development. Further functional analysis of the key gut microbes, metabolites, and genes highlighted the importance of the vitamin B6 metabolism pathway in the process of gut microbiome dysbiosis-induced HUA.

**Figure 9:**
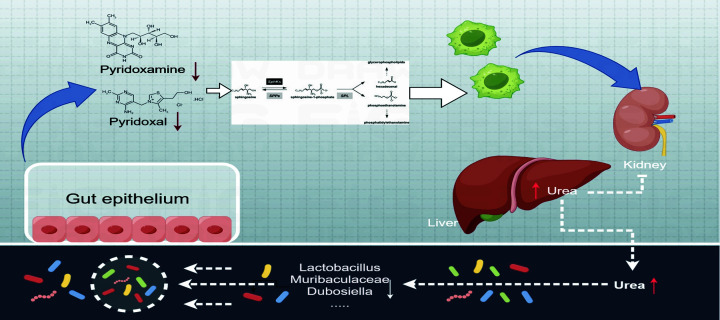
Regulatory role of gut microbiota in hyperuricemia by modulating vitamin B6 metabolism and sphingolipid-driven inflammation As shown, reduced abundance of GM genera such as *Lactobacillus, Muribaculaceae*, and *Dubosiella* altered the pH and led to the production of HUA-inducing metabolites and decreased the levels of pyridoxamine and pyridoxal. The reduction of pyridoxamine and pyridoxal down-regulated sphingosine 1-phosphate lyase activity and decreased the accumulation of sphingosine 1-phosphate. Dysregulation of vitamin B6 metabolism and sphingolipid metabolism promotes inflammation. Excessive levels of inflammatory cytokines in the body cause elevated levels of uric acid.

Gout is not only an autoinflammatory disease, but also a metabolic disorder [47]. Vitamin B6 is essential for sustaining normal metabolic and immune functions, especially modulation of anti-inflammatory responses [48,49]. Specifically, vitamin B6 directly reduces excessive inflammation by decreasing the levels of sphingosine 1-phosphate (S1P) through upregulation of sphingosine 1-phosphate lyase activity [49]. S1P is a signaling lipid molecule that regulates many essential processes, including adaptive immunity and innate immunity [50]. In the present study, our data demonstrated significant reduction of pyridoxamine, pyridoxal, and sphinganine levels in the HUA model group compared with the control group. On the basis of previous reports and our present study, we hypothesized that GM dysbiosis causes gout and HUA by modulating vitamin B6 metabolism through lowering of pyridoxamine and pyridoxal levels, which subsequently activates sphingolipid metabolism directly and leads to excessive inflammation (as summarized in Figure 9). Overall, our research demonstrated that the GM regulated the development of HUA by modulating vitamin B6 metabolism.

Collectively, these results demonstrate the impact of a dysbiotic gut microbiome and the changes in both bacterial and host metabolism on the pathogenesis of gout and HUA. Furthermore, this study unraveled the critical links between HUA-related changes with vitamin B6 metabolism and the associated host inflammatory response.

This study has a few limitations. Firstly, some of the associations require further experimental confirmation. Secondly, we used an untargeted metabolomics approach, which measured relative abundances rather than the absolute concentrations of metabolites. Lastly, even though our 16S rRNA sequencing data identified significant changes in genus of *Bacteroides, Alloprevotella, Parabacteroides, Lactobacillus, Lachnospiraceae_NK4A136_group, Clostridia_UCG_014, Muribaculaceae*, and *Dubosiella* in the HUA microbiome, the individual functions of these microbiota were not assessed. Further studies are necessary to understand the role of these gut microbes in the production of specific metabolites and HUA pathophysiology. Therefore, our data provide significant information that is beneficial for the identification of clinical biomarkers and therapeutic targets for HUA.

## Conclusions

Our study demonstrated the existence of causal relationships between GM, immune cells, and gout. Furthermore, this study demonstrated that the development of HUA and gout involved regulation of vitamin B6 metabolism by the GM through lowering of pyridoxamine and pyridoxal levels, which subsequently modulated sphingolipid metabolism to directly activate excessive inflammation and suppress CD16 on CD14^−^CD16^+^ monocytes.

## Supplementary Material

Supplementary Tables S1-S6

## Data Availability

All data generated or analysed during this study are included in this published article and its supplementary information files.

## Abbreviations

AUC: area under the curve
DEGs: differentially expressed genes
ESI+: electrospray ionization positive mode
ESI−: electrospray ionization negative mode
FC: fold changes
FDR: false discovery rate
GM: gut microbiota
GO: gene ontology
GWAS: genome-wide association studies
HUA: hyperuricemia
IVs: instrumental variables
IVW: inverse variance weighted
KEGG: Kyoto Encyclopedia of Genes and Genomes
LDA: Linear discriminant analysis
LefSe: LDA effect size
MDA: mean decrease in accuracy
MetPA: metabolomics pathway analysis
MR: Mendelian randomization
NLRP3: NLR family pyrin domain containing 3
O2-PLS: prthogonal projections to latent structures by means of partial least squares
OPLS-DA: orthogonal partial least squares discriminant analysis
PCA: principal component analysis
PCoA: principle-coordinates analysis
PICRUSt2: phylogenetic investigation of communities by reconstruction of unobserved states 2
PLS-DA: partial least squares discriminant analysis
PO: potassium oxonate
RF: random forest
RNA-seq: sequencing of RNA
ROC: receiver operating characteristic
S1P: sphingosine 1-phosphate
SCFAs: short-chain fatty acids
SNPs: single nucleotide polymorphisms
SPF: specific pathogen free
UA: uric acid
UHPLC/Q-Orbitrap-MS: ultra-high performance liquid chromatography coupled with quadrupole-orbitrap mass spectrometry
VIP: variable importance in projection
XDH: xanthine dehydrogenase
